# MALAT1 expression in granulosa cells in PCOS patients with different phenotypes

**DOI:** 10.1038/s41598-024-55760-9

**Published:** 2024-02-29

**Authors:** Shumin Li, Yimeng Li, Xueqi Yan, Shigang Zhao, Ziyi Yang, Yuteng Wang, Xueying Gao, Zi-Jiang Chen

**Affiliations:** 1 Department of Reproductive Medicine, Ren Ji Hospital, Shanghai Jiao Tong University School of Medicine, Shanghai, China; 2 Shanghai Key Laboratory for Assisted Reproduction and Reproductive Genetics, Shanghai, China; 3 Institute of Women, Children and Reproductive Health, Shandong University, Jinan, 250012 China; 4 National Research Center for Assisted Reproductive Technology and Reproductive Genetics, Shandong University, Jinan, China; 5 State Key Laboratory of Reproductive Medicine and Offspring Health, Shandong University, Jinan, 250012 Shandong China; 6 Gusu School, Nanjing Medical University, Nanjing, Jiangsu China

**Keywords:** Polycystic ovary syndrome, MALAT1, Long noncoding RNA, Granulosa cells, Metabolism, Endocrine reproductive disorders, Endocrine reproductive disorders

## Abstract

Polycystic ovary syndrome (PCOS) is one of the most common reproductive endocrine metabolic disorders. The lncRNA metastasis-associated lung adenocarcinoma transcript 1 (MALAT1) reportedly can regulate the reproductive system. Despite studies, the alteration of *MALAT1* expression in granulosa cells (GCs) from PCOS patients was inconsistent. To evaluate *MALAT1* expression in GCs in different PCOS subgroups and its association with PCOS phenotypes, we collected GCs from 110 PCOS cases and 71 controls, and examined *MALAT1* expression by quantitative PCR. The results showed *MALAT1* expression was upregulated in PCOS cases, especially in insulin resistant (IR) PCOS subgroup, obese PCOS subgroup and non-hyperandrogenic (NHA) PCOS subgroup. *MALAT1* expression was positively correlated with BMI and several metabolic parameters in controls. Interestingly, *MALAT1* expression was notably associated with some critical endocrine indexes for PCOS, including E2, FSH, LH and LH/FSH ratio. In different PCOS subgroups, we found significant positive correlations with LH/FSH ratio in IR-PCOS and PCOS with normal weight, and with serum T and LH level in NHA-PCOS subgroup. Integrated analysis with lncRNA target databases and PCOS-related databases revealed MALAT1 could participate in PCOS by influencing immune response and lipids metabolism in GCs. In conclusion, MALAT1 was differently expressed in GCs in PCOS, especially in IR, obese and NHA PCOS subgroups. MALAT1 was likely involved in metabolism and immune response in GCs in PCOS. However, more studies are necessary to establish this concept.

## Introduction

Polycystic ovary syndrome (PCOS) is a common reproductive endocrine metabolic disorder in women of reproductive age, affecting 5–18% of women^1,2^. Clinical manifestations of PCOS are heterogenous, characterised by excessive androgens, ovulatory dysfunction and polycystic ovarian morphology. Due to the complexity of PCOS, its etiology needs further exploration, which, with advances in research, has suggested that PCOS development partially depends on noncoding RNAs and epigenetic factors^3^. Ovarian granulosa cells (GCs), are the main site of steroid hormone synthesis in females, and can be involved in the formation of the follicular microenvironment and the growth and development of oocytes^4,5^. The disturbance of cellular biology and gene expression in GCs is implicated in the development of PCOS^6^.

Long noncoding RNAs (lncRNAs) consist of 200–800 nucleotides and, despite lacking protein-coding capacity, play vital roles in various cellular activities and human diseases^7,8^. Metastasis-associated lung adenocarcinoma transcript 1 (MALAT1) is a conserved lncRNA in humans and was initially identified to be upregulated in human lung cancer^9^. Recently, a few studies have focused on its role in reproductive system, like pregnancy process and PCOS^10–14^. While studies have shown differential expression of MALAT1 in GCs and peripheral blood from PCOS patients and PCOS-model rats^7,10–14^, how MALAT1 expression is altered in GCs is inconsistent across studies due to small research cohorts^11,13,14^. Apart from this, the alterations of MALAT1 expression in GCs in PCOS and in different PCOS subtypes are still unclear.

In our study, we aimed to investigate the changes in MALAT1 expression in GCs by studying a larger cohort of patients, including different subtypes of PCOS. Additionally, we explored the relationship between MALAT1 expression and various clinical parameters, providing the theoretical foundation for clarifying the pathophysiology of PCOS.

## Materials and methods

### Participants and ethical statement

A total of 181 Chinese women participated in this study, including 110 patients with polycystic ovary syndrome (PCOS) and 71 controls. From October 2015 to June 2016, ovarian granulosa cells were collected from participants undergoing treatment at the Center for Reproductive Medicine, Shandong University. PCOS was diagnosed according to the Rotterdam criteria^15^, requiring the presence of at least two among the following: (1) clinical and/or biochemical indications of hyperandrogenism; (2) polycystic ovaries, the presence of 12 or more follicles measuring 2–9 mm in diameter in each ovary and/or an ovarian volume of more than 10 mL as determined by ultrasound; (3) oligo- or anovulation, menstrual cycle length of less than 21 or more than 35 days, and/or fewer than eight cycles per year. Participants with other etiologies such as androgen-secreting tumors, Cushing’s syndrome, or congenital adrenal hyperplasia and other hypothalamus, pituitary gland, and ovaries disorders were excluded. Control participants had regular menstrual cycles (26–35 days), normal steroid hormone levels, and normal ovarian morphology. Control participants visited the IVF center because of oviduct and/or male factors related infertility. The long gonadotropin-releasing hormone agonist protocol was applied for participants’ treatment.

This study was approved by Ethics Committee of Hospital for Reproductive Medicine Affiliated to Shandong University and Ethics Committee of Shandong University (No. 2014020) on March 4th, 2014. As well, all experiments involving human patients were in line with the 1964 Helsinki declaration and its later amendments or comparable ethical standards. All participants provided informed consent. All experimental protocols were conducted in line with the respective guidelines and regulations approved by the Institutional Review Board of Shandong University.

### Ovarian stimulation and granulosa cells (GCs) sampling

All participants who underwent the long gonadotropin-releasing hormone agonist protocol received injections of gonadotropin-releasing hormone agonists at the onset of the mid-luteal phase, and ultrasound scans of follicular development and serum estradiol measurements were performed at 1- to 3-day intervals. When more than 3 follicles were measuring ≥ 18 mm in diameter, an appropriate amount of human chorionic gonadotropin (hCG) was then injected. Thirty-six hours after the hCG injection, oocytes were retrieved under ultrasound guidance. GCs from each participant were derived from several pooled follicles and collected from approximately 40–50 mL of follicular fluid using sterile tubes and isolated using Ficoll-Percoll (Solarbio-Life-Sciences, Beijing, China) as previously described^16^.

### RNA extraction and quantitative real-time polymerase chain reaction (qRT-PCR)

Total RNA extraction was conducted from GCs isolated from approximately 40–50 ml of follicular fluid using TRIzol Reagent (Takara Bio, Inc., Dalian, China) as recommended by the manufacturer. For each sample, a total of 1 μg extracted RNA was transcribed into cDNA after treatment with Prime Script RT reagent Kit with gDNA Eraser (Takara Bio, Inc., Dalian, China). The subsequent qRT-PCR analysis used SYBR Premix Ex Taq (Takara Bio, Inc., Dalian, China) and a LightCycler 480 system. There were four stages in qPCR. The first stage (initial denaturation): the reaction temperature was 95 °C for 30 s. The second stage (PCR): the reaction temperature was 95 °C for 5 s and then 60 °C for 30 s. This stage was set at 40 cycles. The third stage (melting): the reaction temperature was 95 °C for 5 s and then 65 °C for 60 s. The fourth stage (cooling): the reaction temperature was 50 °C for 30 s and then 4 °C. The normalization was undertaken to the housekeeping gene *Actin*, and the 2^−ΔCt^ method was applied for *MALAT1* relative expression calculation.

Forward primer sequence for *Actin* was TTCGAGCAAGAGATGGCCA (5′–3′), and reverse primer sequence for *Actin* was CGTACAGGTCTTTGCGGATG (5′–3′). Forward primer sequence for *MALAT1* was AAAGCAAGGTCTCCCCACAAG, reverse primer sequence for *MALAT1* was GGTCTGTGCTAGATCAAAAGGCA (5′–3′).

### Bioinformatic prediction and integrated analysis with public database

PCOS related genes were downloaded by searching with Associated Phenotypes “PCO (polycystic ovarian syndrome)” from Ovarian Kaleidoscope Database (OKdb) (https://appliedbioinfo.com/4_search.html) (Supplementary Table S1). PCOS related miRNAs list was downloaded from Knowledgebase on Polycystic Ovary Syndrome (PCOSKB) (http://pcoskb.bicnirrh.res.in/mirna.php?page=0#) (Supplementary Table S2). MALAT1 target gene list was downloaded from LncRNA2Target (http://123.59.132.21/lncrna2target/index.jsp) (Supplementary Table S3). MALAT1 target gene list from LncSEA (https://bio.liclab.net/LncSEAv2/download.php) was downloaded and filtered in RNA binding protein class (Supplementary Table S4). MALAT1 target miRNAs list from LncSEA was downloaded and filtered in MicroRNA class (Supplementary Table S5). We compared MALAT1 target genes and target miRNAs with PCOS related genes and miRNAs separately.

### Study design and statistical analyses

The threshold for defining insulin resistance is homeostasis model assessment for insulin resistance (HOMA-IR) > 2.5. The threshold for defining hyperandrogenemia is serum total testosterone (T) > 48.1 ng/dL. The threshold for defining obesity is body mass index (BMI) > 25 kg/m^2^.

Data was analysed using R software (version 4.2.1). Spearman was used for correlation analysis. *P* < 0.05 was statistically significant. Our data were presented as the mean ± standard deviation (SD). Student’s t-test was performed to analyse two different groups, whereas one-way ANOVA followed by Tukey’s test was performed for multiple comparisons. Figures were generated using ggplot2 R package (version 3.4.2).

## Results

### Baseline characteristics

We compared the baseline characteristics of 110 PCOS cases and 71 controls enrolled in our study in Table 1. The age range of all participants was 20–38 years old. Participants with PCOS had a significantly higher BMI compared to the control group (*p* < 0.001***). PCOS cases also had worse glucose and lipid metabolism profile, characterised by higher level of FPG (*p* < 0.001***), FINS (*p* < 0.001***), HOMA-IR (*p* < 0.001***), TC (*p* < 0.05*) and TG (*p* < 0.001***). As expected, serum LH (*p* < 0.001***), LH/FSH ratio (*p* < 0.001***), TT (*p* < 0.001***) and AMH (*p* < 0.001***) was significantly higher in PCOS patients compared to controls, but serum FSH (*p* < 0.01**) was lower.

**Table1:** The baseline characteristics of participants.

	Ctrl	PCOS	*p*
n	71	110	
Age	28.56 (2.95)	28.55 (3.33)	0.985
BMI (kg/m^2^)	21.92 (3.02)	24.88 (4.21)	< 0.001***
Metabolic profile			
FPG (mmol/L)	5.15 (0.48)	5.43 (0.51)	< 0.001***
FINS (mIU/L)	7.89 (1.96)	16.82 (10.76)	< 0.001***
HOMA-IR	1.81 (0.49)	4.13 (2.79)	< 0.001***
TC (mmol/L)	4.28 (0.78)	4.49 (0.62)	0.039*
TG (mmol/L)	0.89 (0.40)	1.20 (0.52)	< 0.001***
Hormone profile			
FSH (U/L)	6.59 (1.22)	5.89 (1.46)	0.001**
LH (IU/L)	5.22 (1.68)	8.87 (4.73)	< 0.001***
LH/FSH	0.81 (0.27)	1.61 (1.12)	< 0.001***
E2 (pg/ml)	35.00 (11.14)	38.75 (17.17)	0.105
TT (ng/dl)	23.56 (7.66)	40.80 (17.31)	< 0.001***
AMH (ng/ml)	4.83 (3.17)	9.87 (5.07)	< 0.001***

Clinical baseline characteristics of the control, PCOS and subgroups, including age, body mass index (BMI), metabolic parameters and hormonal parameters. Values are expressed as mean (standard deviation).

*FPG* fasting plasma glucose, *FINS* fasting insulin, *HOMA-IR* homeostasis model assessment for insulin resistance, *TC* total cholesterol, *TG* total glycerol, *FSH* follicle stimulating hormone, *LH* luteinizing hormone, *E2* estradiol, *TT* total testosterone, *AMH* anti-Müllerian hormone.

*p < 0.05, ** *p* < 0.01, *** *p* < 0.001 versus Control group.

### MALAT1 expression in granulosa cells of controls and PCOS cases

**Figure 1:**
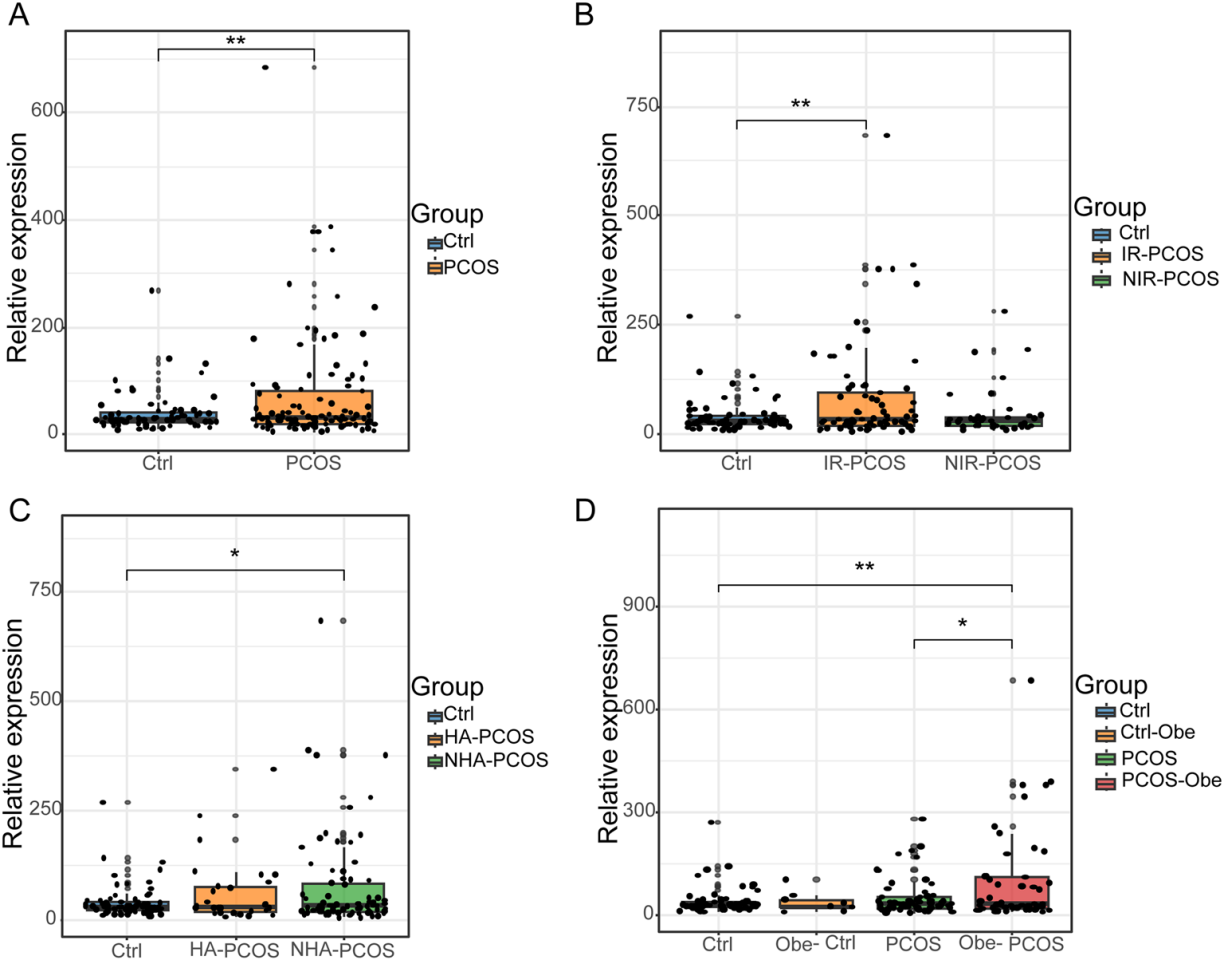
MALAT1 expression was increased in GCs from PCOS, especially IR, NHA-PCOS and Obese subgroups. Data were normalized by *Actin*. (**A**) The relative expression of MALAT1 in PCOS cases (n = 110) and control women (n = 71). (**B**) The normalized expression of MALAT1 in IR-PCOS cases (n = 72), NIR-PCOS cases (n = 38) and control women (n = 71). (**C**) The normalized expression of MALAT1 in HA-PCOS cases (n = 31), NHA-PCOS cases (n = 79) and control women (n = 71). (**D**) The normalized expression of MALAT1 in PCOS cases with normal weight (n = 61), obese PCOS cases (n = 49), control women with normal weight (n = 62) and obese control women (n = 9).

Firstly, we extracted RNA in GCs and performed qRT-PCR to detect the expression level of *MALAT1*. We found a remarkable increase of *MALAT1* expression in PCOS cases (*p* < 0.01**, Fig. 1A). To study the relationship of *MALAT1* expression with other classical PCOS phenotypes, we further clarified PCOS cases into IR-PCOS and NIR-PCOS subtypes according to HOMA-IR index. We found that only IR-PCOS rather than NIR-PCOS patients showed *MALAT1* expression upregulation compared to controls (*p* < 0.01**, Fig. 1B). Moreover, PCOS patients were divided into HA-PCOS and NHA-PCOS subgroups depending on the presence of hyperandrogenism or not. Their comparison result showed the upregulation of *MALAT1* in NHA-PCOS cases compared to controls (*p* < 0.05*, Fig. 1C). Considering that PCOS was often associated with obesity, we also focused on the *MALAT1* expression difference between controls and PCOS cases with normal weight and obesity (Obe). We noticed that the expression of *MALAT1* expression in Obe-PCOS GCs was higher not only than controls (*p* < 0.01**, Fig. 1D), but also than PCOS with normal weight (*p* < 0.05*, Fig. 1D). The results of this part illustrated that abnormal metabolism, obesity and abnormal androgen were associated with *MALAT1* expression in GCs.

### Association of the MALAT1 expression with clinical characteristics

**Figure 2:**
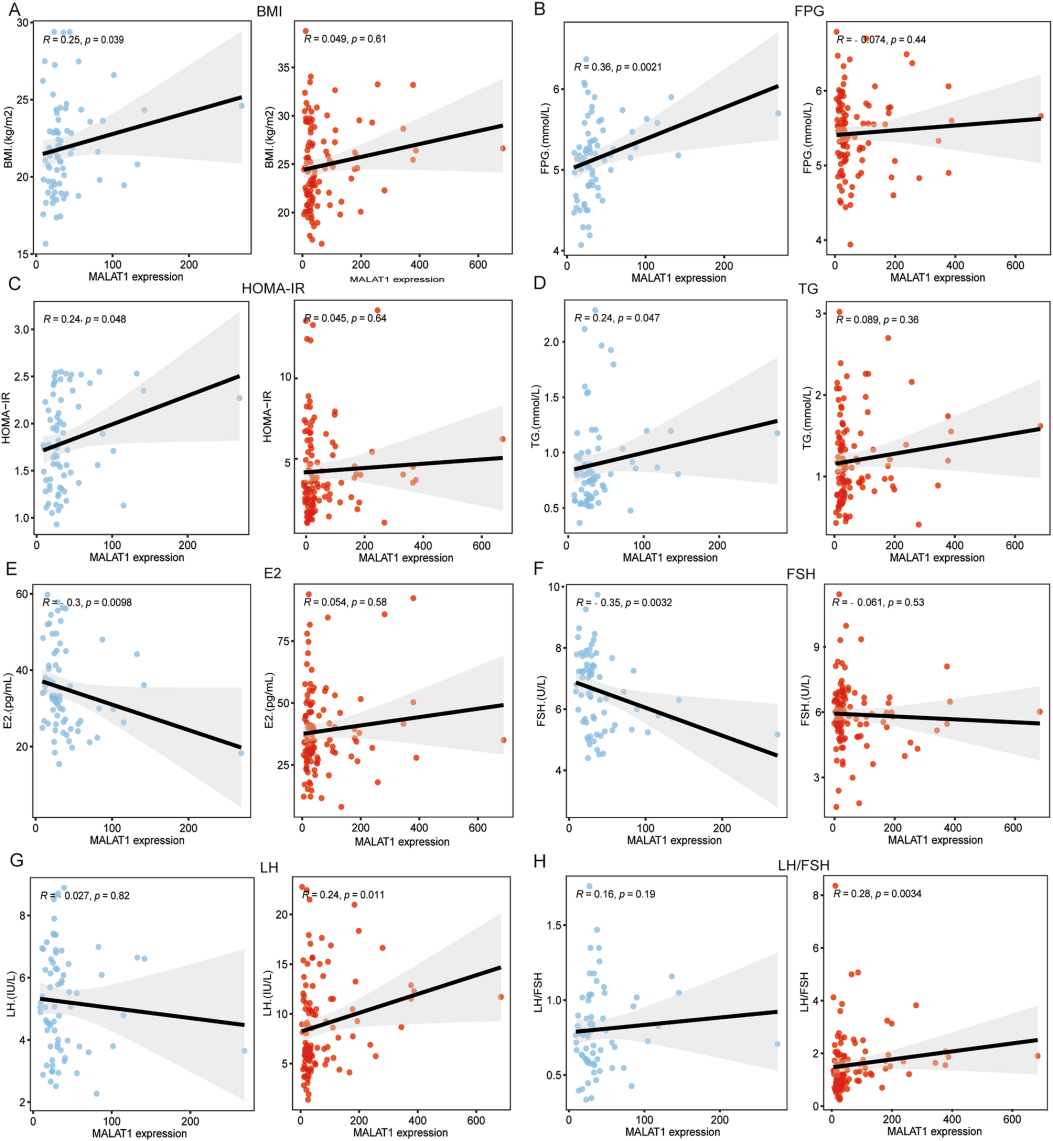
MALAT1 expression was correlated with some clinical characteristics in controls and PCOS cases. (**A**–**H**) The relationships of *MALAT1* expression and BMI, FPG, HOMA-IR, TG, E2, FSH, LH and LH/FSH ratio in control group and PCOS group. Statistical analysis of the data was performed using Spearman’s test. The blue dots represented control group and the red dots represented PCOS group.

We compared correlation results of *MALAT1* expression and clinical characteristics in PCOS and control groups using Spearman’s correlation. Interestingly, the correlation analysis showed that the expression of *MALAT1* was remarkedly positively correlated with BMI (*R* = 0.25, *p* = 0.039*; Fig. 2A) only in controls. Constantly, there were positive correlations between the expression of *MALAT1* and metabolic parameters only in controls as well, including FPG (*R* = 0.36, *p* = 0.0021*; Fig. 2B), HOMA-IR (*R* = 0.24, *p* = 0.048*; Fig. 2C) and TG (*R* = 0.24, *p* = 0.047*, Fig. 2D).

As for endocrine profiles, we observed the significant correlations between the *MALAT1* expression and several important hormone indexes for PCOS. *MALAT1* expression was significantly correlated with serum E2 level (R = -0.3, *p* = 0.0098**, Fig. 2E) and serum FSH level (R = − 0.35, *p* = 0.0032**, Fig. 2F) only in controls. Besides, we observed the significant positive correlations between *MALAT1* expression and serum LH level (R = 0.24, *p* = 0.011*, Fig. 2G) and LH/FSH ratio (R = 0.28, *p* = 0.0034**, Fig. 2H) in PCOS group. We suggested that MALAT1 in GCs could take part in endocrine regulation in female.

### Association of the MALAT1 expression with clinical characteristics in different PCOS subgroups

**Figure 3:**
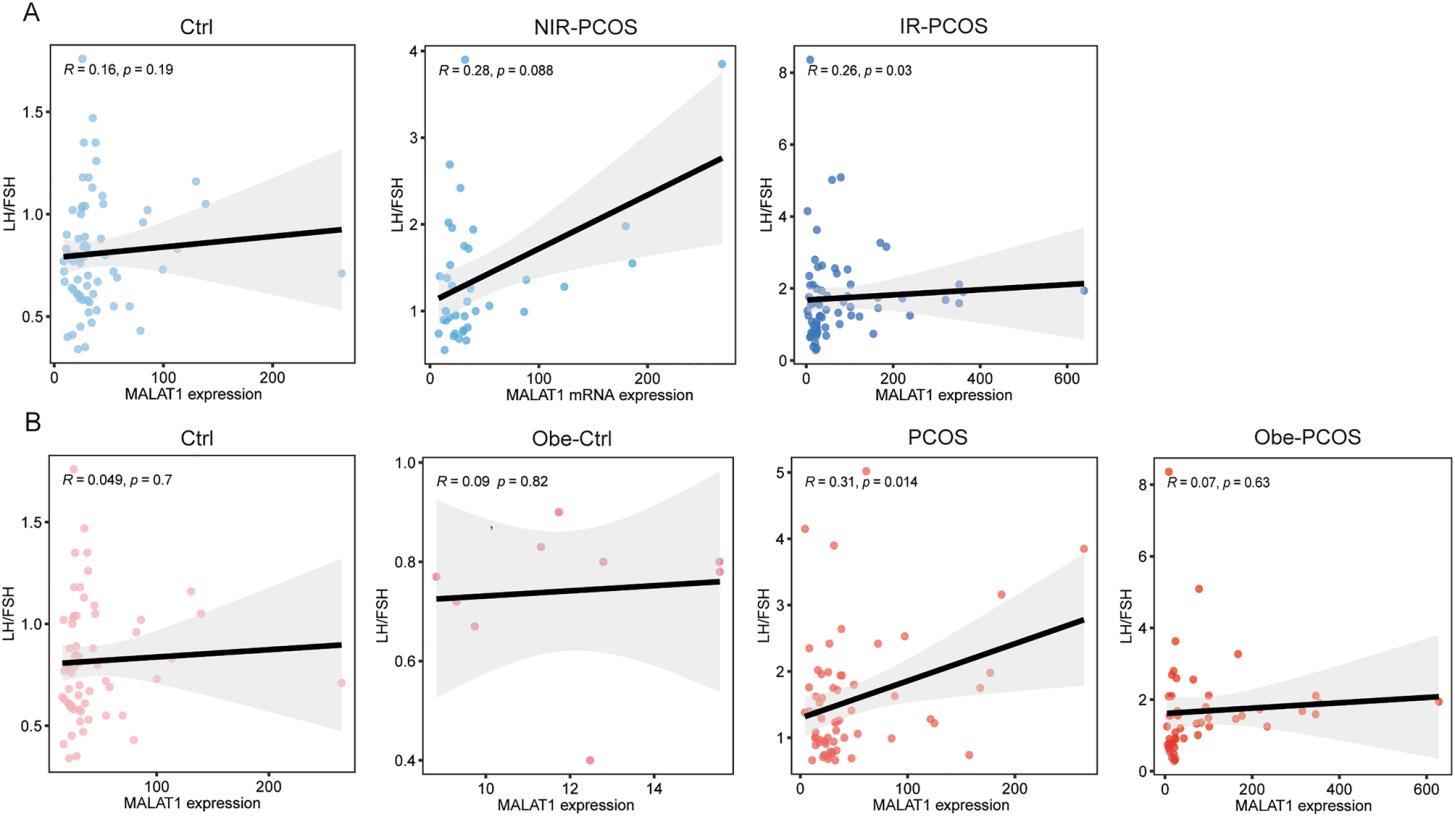
MALAT1 expression was correlated with LH/FSH ratio in different PCOS subgroups. (**A**) The relationship of MALAT1 expression and LH/FSH ratio in controls, NIR-PCOS and IR-PCOS cases. (**B**) The relationship of MALAT1 expression and LH/FSH ratio in controls, obese controls, PCOS and obese PCOS cases. Statistical analysis of the data was performed using Spearman’s test.

Next, we further detected the association of the *MALAT1* expression with clinical characteristics in different PCOS subgroups. Firstly, we compared the associations amongst controls, NIR-PCOS and IR-PCOS subgroups. Results showed the significant correlations between the *MALAT1* expression and serum LH/FSH ratio (R = 0.26, *p* = 0.03*, Fig. 3A) only in IR-PCOS group.

Besides, the serum LH/FSH ratio was significantly positively correlated with *MALAT1* expression (R = 0.31, *p* = 0.014*, Fig. 3B) in PCOS women with normal weight when we detected the associations amongst controls, obese controls, PCOS and obese PCOS cases.

**Figure 4:**
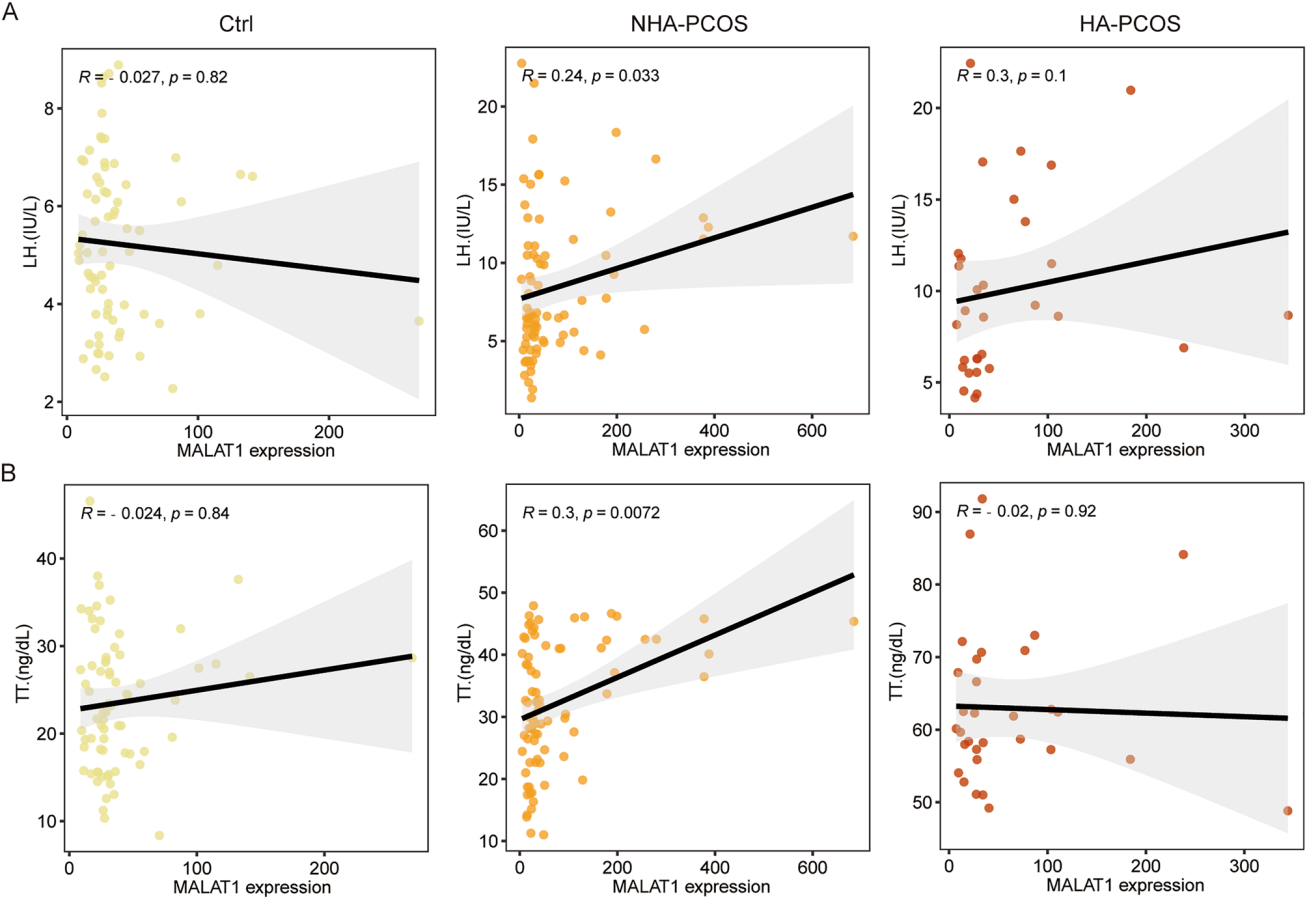
MALAT1 expression was correlated with some clinical characteristics in controls, NHA-PCOS and HA-PCOS cases. (**A**) The relationship of MALAT1 expression and serum LH level in controls, NHA-PCOS and HA-PCOS cases. (**B**) The relationship of MALAT1 expression and serum TT level in controls, obese controls, PCOS and obese PCOS cases. Statistical analysis of the data was performed using Spearman’s test.

We then explored the associations amongst controls, NHA-PCOS and HA-PCOS subgroups. Interestingly, we found the significant positive correlations between *MALAT1* expression and serum LH level (R = 0.24, *p* = 0.033*, Fig. 4A) and serum T level (R = 0.3, *p* = 0.0072**, Fig. 4B) solely in NHA-PCOS subgroup.

### Regulation network of MALAT1 by integrated analysis with public database

**Figure 5:**
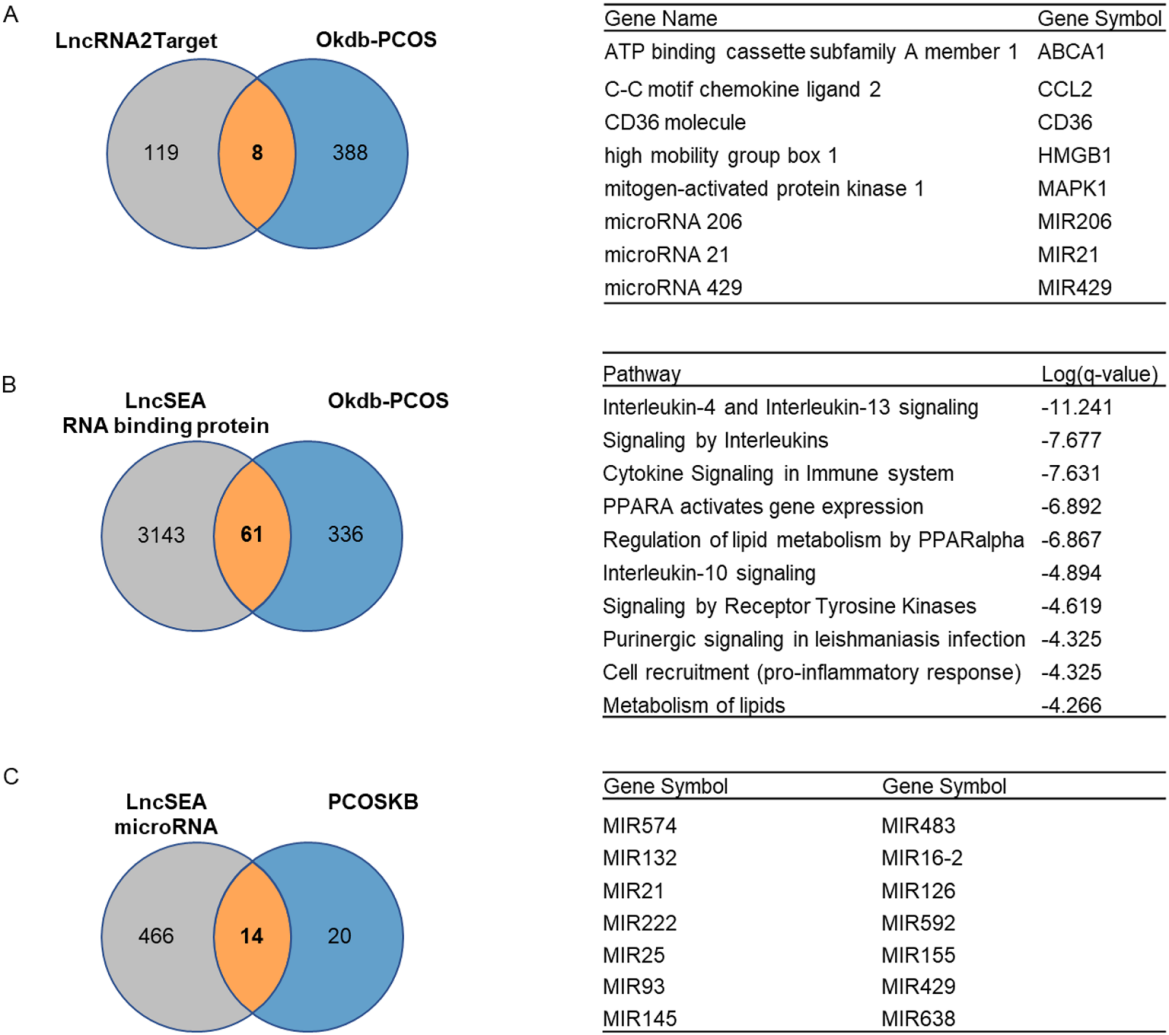
Regulation network of MALAT1 was generated by integrated analysis with public database. (**A**) Overlapped genes of MALAT1 target genes in LncRNA2Target and PCOS-related genes in OKdb. (**B**) Overlapped genes of MALAT1 target genes in RNA binding protein term in LncSEA and PCOS-related genes in OKdb, and these genes enrichment according to Reactome gene set. (**C**) Overlapped miRNAs of MALAT1 target miRNAs in microRNA term in LncSEA and PCOS-related miRNAs in PCOSKB.

Aiming to explore the biological function of MALAT1 in PCOS, we integrated two critical lncRNA databases, LncSEA (http://bio.liclab.net/LncSEA/index.php) and LncRNA2Target (http://123.59.132.21/lncrna2target/index.jsp) with PCOS related Ovarian Kaleidoscope Database (OKdb) and Knowledgebase on Polycystic Ovary Syndrome (PCOSKB) databases. First of all, referring to the PCOS related genes in Ovarian Kaleidoscope Database (OKdb) and LncRNA2Target, there were 8 overlapped genes, including ABCA1, CCL2, CD36, HMGB1, MAPK1, MIR206, MIR21 and MIR429 (Fig. 5A). Moreover, when referring to PCOS related genes in OKdb and RNA binding protein term in LncSEA, there were 61 overlapped genes (Fig. 5B, left). We then enriched these genes into Reactome gene set, and found they were mainly involved in immune pathways, like *Interleukin-4 and Interleukin-13 signaling*, *Signaling by Interleukins* and *Cytokine Signaling in Immune system* (Fig. 5B, right).

**Figure 6:**
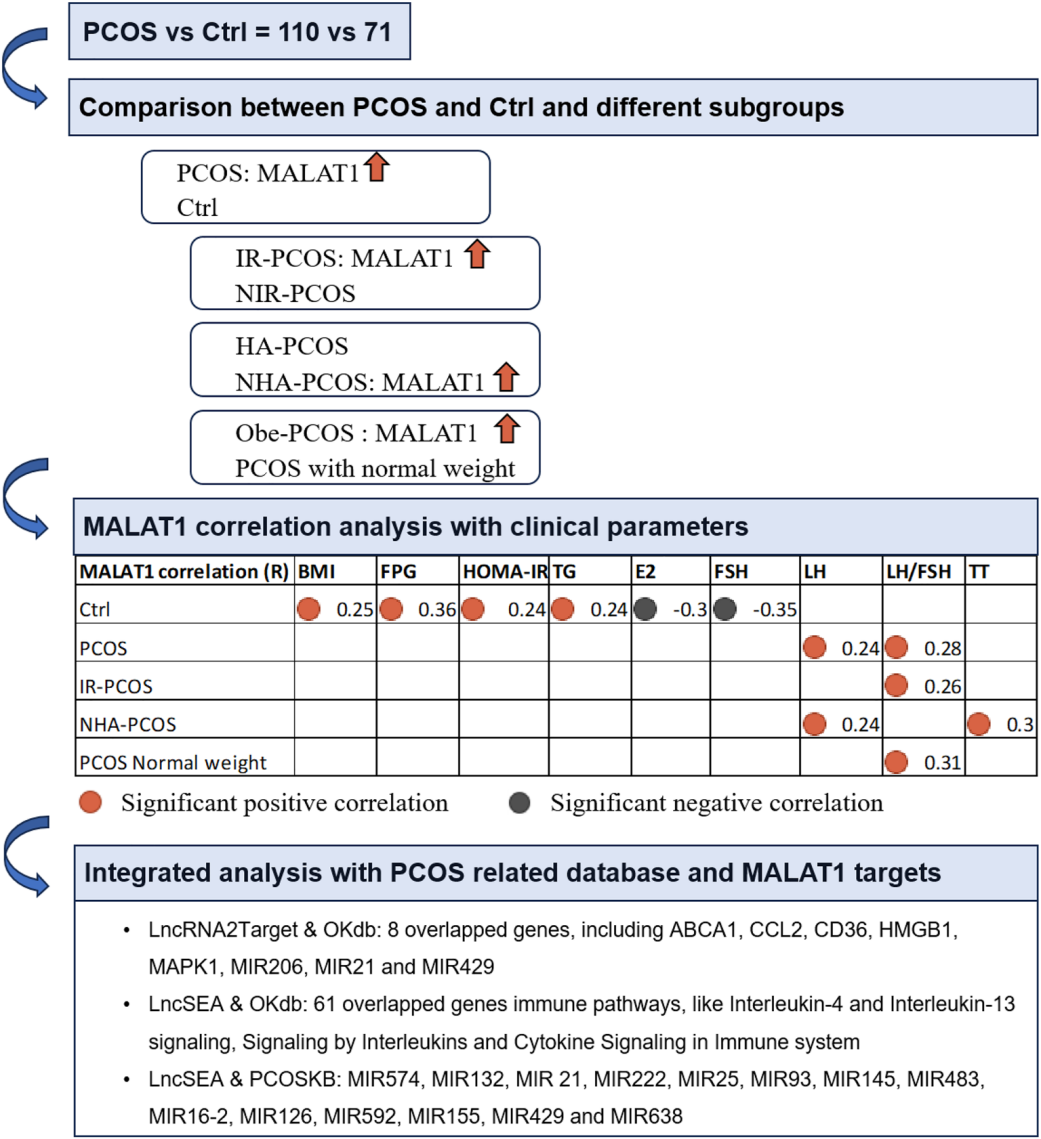
Graphical summary.

MALAT1 can also regulate various miRNAs expression. Combining microRNA term in LncSEA with PCOS related miRNA database PCOSKB (http://pcoskb.bicnirrh.res.in/), we observed 14 overlapped miRNAs, including MIR574, MIR132, MIR 21, MIR222, MIR25, MIR93, MIR145, MIR483, MIR16-2, MIR126, MIR592, MIR155, MIR429 and MIR638 (Fig. 5C). Altogether, our research was summarised in Fig. 6.

## Discussions

As a conserved lncRNA, MALAT1 was reported to exert great impact on multiple biological processes^9,17,18^. However, its alteration in GCs in PCOS and its association with PCOS remained unclear yet. In the present study, we systematically evaluated *MALAT1* expression level in GCs in a large cohort, and compared *MALAT1* expression in different PCOS subgroups. We showed that the expression of *MALAT1* was significantly higher in PCOS GCs, partly consistent with the recently published study^14^. In the meanwhile, *MALAT1* was upregulated especially in IR-PCOS, Obe-PCOS and NHA-PCOS subgroups. We found *MALAT1* gene expression was significantly positively correlated with BMI and several metabolic parameters in controls, like FPG, HOMA-IR and TG. Interestingly, *MALAT1* gene expression was also remarkedly associated with a few critical endocrine indexes for PCOS, including serum E2, FSH, LH and LH/FSH ratio. Moreover, further exploration of associations in different PCOS subgroups, we found the significant positive correlation with LH/FSH ratio in IR-PCOS and PCOS with normal weight, and the significant positive correlations with serum T and LH level in NHA-PCOS subgroup. In the end, we integrated MALAT1 targets with public PCOS databases and constructed the regulation network of MALAT1. These findings partially shed light on the roles of MALAT1 during the pathophysiological process of PCOS.

Emerging evidence indicated *MALAT1* expression was altered in PCOS. It was reported that *MALAT1* expression was downregulated in peripheral blood from PCOS patients^7,11^. However, there was divergence on the alterations in PCOS patients’ GCs. Zhao et.al and Tan et.al found lower expression level of *MALAT1* in GCs from PCOS patients^12,13^, while high-throughput sequencing and qRT-PCR results in one study published in 2022 revealed the upregulation of *MALAT1* in PCOS GCs^14^. Moreover, upregulation of AMH, one of the characteristics of PCOS, had the capacity to increase MALAT1 expression in GCs^14^. PCOS cases involved in previous two studies had higher T level, but in our study PCOS cases were clarified into hyperandrogenic and non-hyperandrogenic subgroups^12,13^. In the meanwhile, another study revealing the *MALAT1* upregulation also only included non-hyperandrogenic PCOS patients^14^. Consistently, in our research, we observed the absolute upregulation of *MALAT1* in NHA-PCOS subgroup, and the significant positive correlation of *MALAT1* expression and serum T level in NHA-PCOS patients. Besides, we also found the significant positive correlation of *MALAT1* expression and serum LH level especially in NHA-PCOS patients. Altogether, these results suggested that there might be different pathophysiology involving MALAT1 in GCs in PCOS with or without hyperandrogenism.

Interestingly, in our study we detected the notable correlation between *MALAT1* expression and BMI, FPG, HOMA-IR and TG, but only in controls. Besides, the increase of *MALAT1* was much more obvious in IR-PCOS subgroup. The role of MALAT1 was demonstrated in some metabolic disorders, like obesity and diabetes^19,20^. Preliminary research on obese and normal-weight female participants found that *MALAT1* expression in adipose tissue was significantly positively correlated with HOMA-IR^19^. Moreover, positive correlations were also found between *MALAT1* gene expression and some key lipid metabolism genes, including PPARγ, PGC1, SREBP-1c, FASN, and ACC in adipose tissue^19^. Similarly, when referring to MALAT1 target genes and PCOS related genes, the common genes were also enriched in some lipid metabolism pathways, like *PPARA activates gene expression*, *Regulation of lipid metabolism by PPARalpha* and *Metabolism of lipids*. These results suggested that MALAT1 affected GCs widely in PCOS, as a complex endocrine and metabolism disorder. Except for the extensively-investigated role of MALAT1 in controlling GCs proliferation^11–14^, it was necessary to switch the focus onto its role on metabolism in GCs in the field of PCOS.

As for the signaling pathway affected by MALAT1 in GCs, one research showed that *MALAT1* knockdown in GCs could inhibit the phosphorylation of SMAD2/3, one of the most important mediators in immune response^13^. Consistent with our joint analysis with external databases, MALAT1 may indeed function in GCs by regulating immunity. However, in PCOS model rats, *MALAT1* expression was shown to be downregulated in ovarian tissue of PCOS rats. It was found that overexpression of *MALAT1* seemed to play a protective role in reducing ovarian tissue damage and endocrine disorder in PCOS by regulating miR-302d-3p mediated leukemia inhibitory factor activity^10^. Although the effect of MALAT1 was controversial with our findings for its undertaken in rats, it revealed the critical role of MALAT1 in the immune regulation. Moreover, it was reported that MALAT1 contributed to the pathophysiological process of PCOS by regulating TGFβ signaling through sponging miR-125b and miR-203a^13^. To sum up, the contribution of MALAT1 in PCOS has been poorly studied, and the specific pathology of MALAT1 on PCOS development needs to be further explored.

Overall, the findings of this study demonstrate the differential expression of *MALAT1* in GCs in PCOS, especially in IR, obese and NHA PCOS subgroups and underscore the potential role of MALAT1 in metabolism and immune response in GCs in PCOS. However, it is important to recognise the limitations of this study. The absence and unavailability of protein samples from these participants enrolled in the research prevented MALAT1 protein analysis, hindering the comprehensiveness of the study. The MALAT1 protein analysis in GCs in PCOS needs to be conducted in the future for the comprehensiveness. Moreover, more studies are needed to further investigate the role of MALAT1 in glucose and lipid metabolism and immune response in PCOS.

To summarise, we have investigated that the *MALAT1* expression in GCs of PCOS patients was notably increased, and the increase was much more significant in the NHA-PCOS, IR-PCOS and obe-PCOS subgroups, indicating that the MALAT1-mediated action might take vital roles for the steroid metabolism, IR and metabolism of PCOS in GCs. Regulation network generated by integrating external resource revealed MALAT1’s potential but important role on lipid metabolism and immune response in GCs in PCOS. This study gave a theorical basis for MALAT1’s role in understanding the underlying mechanism of PCOS.

### Supplementary Information

Supplementary Information.

## Data Availability

The datasets used and/or analysed during the current study are available from the corresponding author on reasonable request.

## Abbreviations

MALAT1: Metastasis-associated lung adenocarcinoma transcript 1
PCOS: Polycystic ovary syndrome
lncRNAs: Long noncoding RNAs
GCs: Granulosa cells
hCG: Human chorionic gonadotropin
PCR: Polymerase chain reaction
IR: Insulin resistant
HA: Hyperandrogenic
NHA: Non-hyperandrogenic
BMI: Body mass index
FPG: Fasting plasma glucose
FINS: Fasting insulin
HOMA-IR: Homeostasis model assessment for insulin resistance
TC: Total cholesterol
TG: Total glycerol
FSH: Follicle stimulating hormone
LH: Luteinizing hormone
E2: Estradiol
TT: Total testosterone
AMH: Anti-Müllerian hormone
OKdb: Ovarian Kaleidoscope Database
PCOSKB: Knowledgebase on Polycystic Ovary Syndrome
SMAD: Small mother against decapentaplegic
PPARγ: Peroxisome proliferator-activated receptor gamma
PGC1: Peroxisome proliferator-activated receptor gamma coactivator 1 alpha
FASN: Fatty acid synthase
ACC: Acetyl-CoA carboxylase alpha
